# Spontaneous Perforation of Pyometra Presenting as Acute Abdomen and Pneumoperitoneum Mimicking Those of Gastrointestinal Origin

**DOI:** 10.1155/2015/548481

**Published:** 2015-01-05

**Authors:** Takahiro Yamada, Nanako Ando, Naoshi Shibata, Motomu Suitou, Hiroshi Takagi, Kazutoshi Matsunami, Satoshi Ichigo, Atsushi Imai

**Affiliations:** ^1^Department of Obstetrics and Gynecology, Matsunami General Hospital, Gifu 501-6062, Japan; ^2^Department of Surgery, Matsunami General Hospital, Gifu 501-6062, Japan; ^3^Institute of Endocrine-Related Cancer, Matsunami General Hospital, 150-1 Dendai, Kasamatsu, Gifu 501-6062, Japan

## Abstract

Gastrointestinal (GI) perforation accounts for over 90% of acute abdomen and pneumoperitoneum. The presence of pneumoperitoneum secondary to spontaneously perforated pyometra is an interesting yet confusing finding given the absence of gastrointestinal (GI) perforation, because pyometra is more common in postmenopausal women. We report an instructive case of diffuse peritonitis caused by spontaneous perforation of pyometra. A 70-year-old postmenopausal female was admitted to surgical emergency with signs of diffuse peritonitis. After resuscitation, an emergency laparotomy was performed because of suspicion of GI perforation. At laparotomy, about 2,000 mL of purulent fluid was found to be present in peritoneal cavity, while GI tract was intact. A rent with a diameter of 5 mm was found on anterior fundus of uterus. A total abdominal hysterectomy with a bilateral salpingo-oophorectomy was performed. Despite intensive care and a course of antibiotics, the patient died of multiple organ failure resulting from sepsis on postoperative day 16. Our case illustrates the importance of clinical knowledge of acute gynecological diseases, which are not uncommonly encountered by the general surgeon. Moreover, good appreciation of pelvic anatomy and close collaboration with gynecology and GI surgery colleagues is essential as operative intervention is often required.

## 1. Introduction

Pyometra is defined as buildup of pus (purulent material) in the uterine cavity [1, 2]. It is exceedingly unusual gynecologic condition that happens mostly in postmenopausal age group and hardly ever in premenopausal age group. The presence of pneumoperitoneum secondary to spontaneously perforated pyometra is an interesting yet confusing finding given the absence of gastrointestinal (GI) perforation, because pyometra is more common in postmenopausal women. To date, approximately 50 case reports have been reported in English literature [2–5]. Gastrointestinal (GI) perforation accounts for over 90% of acute abdomen and pneumoperitoneum [2–5], and pneumoperitoneum associated with a ruptured pyometra is recognized only in less than 30% of cases [6, 7]. It is noteworthy that, in most of the cases, a diagnosis of a ruptured pyometra is made intraoperatively, where suspected diagnosis is a GI perforation. This paper reports an additional case of spontaneous rupture of pyometra presenting with generalized peritonitis and pneumoperitoneum.

## 2. Case Presentation

**Figure 1:**
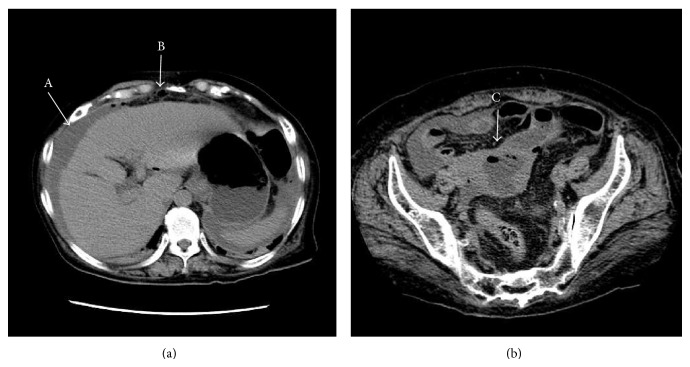
Transverse views of computed tomography showing the presence of fluid (A) and free air (B) in the upper abdomen (a) and a fluid filled uterus and intrauterine free air (C) (b).

A 70-year-old woman with severe abdominal pain and vomiting of 24-hour duration was admitted to our hospital. She had history of scleroderma and rheumatoid arthritis and had been taking steroids for 5 years. In addition, she received operations of thymoma with myasthenia gravis 31 years ago and stomach cancer (pT1AN0M0) 3 years ago. Her gynecological history was unremarkable, and there was no history of postmenopausal bleeding or vaginal discharge. On the physical examination, her abdomen was very tender and was distended and showed muscle rigidity. Rebound tenderness was absent. Bowel sounds were hypoactive. There was no palpable mass. Her blood pressure was 81/43 mmHg and pulse rate was 110 beats/min. Laboratory studies demonstrated a white blood cell count of 22,350 × 10^6^/L and C-reactive protein at 32.39 mg/dL (normal range <0.3). Urgent computed tomography (CT) of the abdomen reported the presence of fluid and free intraperitoneal air mostly in the upper abdomen (Figure 1(a)), in addition to dilatation of upper small intestine. It was not possible to confirm the specific site of perforation accurately but it was suggestive of perforation of GI tract.

Following prompt resuscitation and intravenous antibiotics (flomoxef, 2 g/day, daily administration), laparotomy was performed, which confirmed the presence of free air and pus as well as an inflamed small intestine. Two thousand mL of pus was found to be present in peritoneal cavity. The rest of alimentary tract, gall bladder, and liver were normal. During peritoneal lavage, we found a perforation with a diameter of approximately 5 mm over fundus of the uterus. Total abdominal hysterectomy, copious saline lavage, and tube drainage were performed as a joint procedure. No colonic resection was deemed necessary. The uterus was soft and slightly enlarged. Retrospective review of the CT reported, in addition to the previously documented findings, a fluid-filled uterus with free air in the anterior wall (Figure 1(b)). Histological examination confirmed pyometra with no evidence of malignancy or cervical stenosis. Culture of the pus grew* Bacteroides fragilis*,* Escherichia coli*, and* Eubacterium*.

Postoperatively, intravenous antibiotics (flomoxef, 2 g/day) and sepsis treatment were continued and the patient was admitted to the intensive care unit with strict management of respiration and circulation. Her condition improved over time and she was transferred to the gynecological unit on postoperative day (POD) 4. However, she broke into decrease in blood pressure and saturation, pyrexia, and abdominal distension on POD 11. Although hepatic and renal dysfunctions were not revealed, sepsis and multiple organ failure were suspected. A contrast-enhanced CT showed the increased abdominal pus, and then vancomycin (1 g/day) and meropenem (1 g/day) were added to antibiotic therapy. Despite exhaustive clinical efforts, sepsis grew progressively worse and the patient died on POD 16.

## 3. Discussion

Accumulation of purulent material in the uterine is termed as pyometra. It is an uncommon condition occurring mainly in elderly postmenopausal women and due to impaired drainage of uterine cavity. Impaired drainage conditions include cervical canal stenosis, benign or malignant cervical lesions, and surgical complications [6–9]. Although atrophic endometrium is a common cause of pyometra, perforation is usually seen in the presence of serious causes such as cervical or endometrial carcinoma or a long-term left intrauterine device. Malignant disease is present in 35% of cases [6–9]. The classic triad of pyometra is lower abdominal pain, purulent vaginal discharge, and postmenopausal bleeding, although more than 50% of all cases are asymptomatic [6–9].

Abdominal pain, vomiting, and fever predominate as the presenting symptoms in spontaneously perforated pyometra while gynecological symptoms such as vaginal bleeding or discharge occur in less than 10% [4, 10]. Generalized peritonitis (40–50%) and GI perforation (30–40%) are the most prevalent preoperative diagnoses with radiological findings of pneumoperitoneum present in only half of these [4, 10]. The presence of pneumoperitoneum secondary to spontaneously ruptured pyometra is an interesting yet confusing finding given the absence of GI perforation.

GI perforation is the cause of pneumoperitoneum in 85–95% of cases and requires surgical intervention as its definitive management [10, 11]. Preoperative diagnosis of spontaneously perforated pyometra is difficult but the initial management remains the same as for generalised peritonitis or GI perforation: prompt resuscitation, antibiotics, and radiological investigation. As illustrated by Shapey et al. [10], while CT is often indicated in such circumstances, this case demonstrates that accurate radiological diagnosis of spontaneously perforated pyometra can still be difficult, especially when imaging is reviewed in the acute setting and often not by a specialist abdominopelvic radiologist. Despite the absence of gynecological symptoms in most cases, gynecological sonography may be the most adequate to illustrate fluid accumulation in utero.

The presence of free intraperitoneal and intrauterine air may be due to gas forming organisms such as* Escherichia coli* and* Bacteroides fragilis* [4], although in this case no specific bacterial culture was obtained at laparotomy. The passage of air through the genital canal into the peritoneal cavity is also well documented. Nevertheless, the pathophysiology of spontaneously perforated pyometra is reliant on a closed or stenosed cervix and, together with the absence of any gynecological symptoms, the passage of transcervical air is unlikely in our case.

Ruptured pyometra cases should be managed by total abdominal hysterectomy with bilateral salpingo-oophorectomy, thorough drainage and irrigation of pelvoabdominal cavity, postoperative intensive care support, and administration of broad-spectrum antibiotics [5–7, 12–15]. Mortality from spontaneously performed pyometra exceeds 40% [5–7, 12–15]. It highlights the importance of multidisciplinary involvement in treating sepsis [4–7, 12–15].

In summary, spontaneously perforated pyometra is an interesting yet confusing finding given the absence of gastrointestinal (GI) perforation, because pyometra is more common in postmenopausal women. It should be kept in mind as a differential diagnosis in postmenopausal women presenting with acute abdomen and pneumoperitoneum. An appreciation of acute gynecological disease is essential as many patients with gynecological pathologies may present with signs and symptoms mimicking those of GI origin. Despite the absence of gynecological symptoms in most cases, gynecological sonography may be the most convenient to illustrate fluid accumulation in utero. Correct diagnosis and proper treatment can reduce morbidity and mortality.
